# DHA-Provoked Reduction in Adipogenesis and Glucose Uptake Could Be Mediated by Gps2 Upregulation in Immature 3T3-L1 Cells

**DOI:** 10.3390/ijms241713325

**Published:** 2023-08-28

**Authors:** Natalia Grigorova, Zhenya Ivanova, Ekaterina Vachkova, Valeria Petrova, Toncho Penev

**Affiliations:** 1 Department of Pharmacology, Animal Physiology, Biochemistry and Chemistry, Faculty of Veterinary Medicine, Trakia University, 6000 Stara Zagora, Bulgaria; jen_s@abv.bg (Z.I.); katvach@gbg.bg (E.V.); valeria.petrova1075@gmail.com (V.P.); 2 Department of Ecology and Animal Hygiene, Faculty of Agriculture, Trakia University, 6000 Stara Zagora, Bulgaria; tonchopenev@abv.bg

**Keywords:** docosahexaenoic acid (DHA), cell viability, adipogenesis, lipolysis rate, glucose uptake, Pparg, Gpr120, Gps2, Cpt1, 3T3-L1 cells

## Abstract

The signaling pathway of fatty acids in the context of obesity is an extensively explored topic, yet their primary mechanism of action remains incompletely understood. This study aims to examine the effect of docosahexaenoic acid (DHA) on some crucial aspects of adipogenesis in differentiating 3T3-L1 cells, using palmitic acid-treated (PA), standard differentiated, and undifferentiated adipocytes as controls. Employing 60 µM DHA or PA, 3T3-L1 preadipocytes were treated from the onset of adipogenesis, with negative and positive controls included. After eight days, we performed microscopic observations, cell viability assays, the determination of adiponectin concentration, intracellular lipid accumulation, and gene expression analysis. Our findings demonstrated that DHA inhibits adipogenesis, lipolysis, and glucose uptake by suppressing peroxisome proliferator-activated receptor gamma (Pparg) and G-protein coupled receptor 120 (Gpr120) gene expression. Cell cytotoxicity was ruled out as a causative factor, and β-oxidation involvement was suspected. These results challenge the conventional belief that omega-3 fatty acids, acting as Pparg and Gpr120 agonists, promote adipogenesis and enhance insulin-dependent glucose cell flux. Moreover, we propose a novel hypothesis suggesting the key role of the co-repressor G protein pathway suppressor 2 in mediating this process. Additional investigations are required to elucidate the molecular mechanisms driving DHA’s anti-adipogenic effect and its broader health implications.

## 1. Introduction

Nowadays, obesity is increasing to epidemic proportions, affecting a growing number of young individuals. This complex condition is primarily attributable to genetic predisposition, a sedentary lifestyle, and excessive calorie intake [1]. Maintaining lipid and glucose homeostasis is critical to the body’s energy balance, whereas the functional state of adipose tissue is of pivotal importance [2,3]. Various innovative strategies have been developed to partially reduce the damage caused by daily bad habits, with the role of omega-3 polyunsaturated fatty acids (PUFAs) in preventing obesity and related complications being of significant interest [4,5]. PUFAs, as well-known modulators of whole-body metabolism, are widely used in the therapeutic management of a range of inflammatory diseases and conditions, such as obesity, dermatitis, osteoarthritis, and cardiovascular comorbidities [6,7,8,9,10,11]. Among these fatty acids, docosahexaenoic acid (DHA) is of particular interest since, along with its anti-inflammatory and antiadipogenic features, it seems to have the ability to suppress the proliferation of rapidly dividing cells, making it a promising approach in cancer therapy [12,13,14]. As mentioned above, PUFAs are thought to be a potent modulator of adipocyte metabolism, but the exact mechanism of white adipose tissue’s adaptation to PUFA exposure is poorly understood. PUFAs serve as signaling molecules engaging in a complex interplay with multiple transcription factors and nuclear receptors [5,15,16,17,18]. PUFAs are commonly considered agonists of crucial adipogenic factors such as peroxisome proliferator-activated receptor gamma (Pparg), mediating healthy adipose tissue expansion by enhancing lipogenic gene expression and insulin-dependent glucose uptake while suppressing the lipolysis rate [19,20,21]. Conversely, DHA, despite being a member of the PUFA family, often provokes lipolytic and antiadipogenic effects, along with enhanced mitochondrial biogenesis. This further results in a noteworthy level of weight reduction and suppressed adipocyte proliferation, with many studies even reporting apoptotic effects [22,23,24,25,26,27]. However, in obese individuals, these outcomes may lead to lipotoxicity and trigger adverse health consequences [28,29]. Therefore, when considering dietary supplementation for obese or obesity-predisposed animals and humans, it is crucial to prioritize reducing lipolysis intensity and maintaining the adipose tissue’s buffering capacity rather than pursuing short-term, rapid weight loss.

Recent research has focused extensively on exploring the involvement of G-protein receptors, transcriptional co-activators, and co-suppressors within the complex network of adipocyte biology. They are recognized as the multifunctional factors of various intracellular metabolic pathways, potentially unlocking opposing physiological outcomes depending on the specific signaling interactions. Among them, the G-protein-coupled receptor 120 (Gpr120) [30,31] and the G protein pathway suppressor 2 (Gps2) [32,33] have emerged as prominent regulators in terms of determining the adaptive capacity of adipose tissue in response to high-energy intake. However, the precise role and underlying mechanisms through which DHA influences adipocyte metabolism and interacts with these factors remain poorly understood and warrant further investigation.

The objective of the current study was to investigate the intricate modulation of adipogenesis in vitro caused by DHA supplementation, including intracellular lipid deposition, adiponectin production, cell viability, glucose uptake, lipolysis rate, and the expression of some critical genes involved in adipocyte metabolism. Thus, we aimed to contribute to a broader understanding of the convoluted DHA interplay within adipocyte signaling pathways and to provide insights into its potential therapeutic applications regarding the onset of obesity.

## 2. Results

### 2.1. Pre-Experimental Procedure

**Figure 1:**
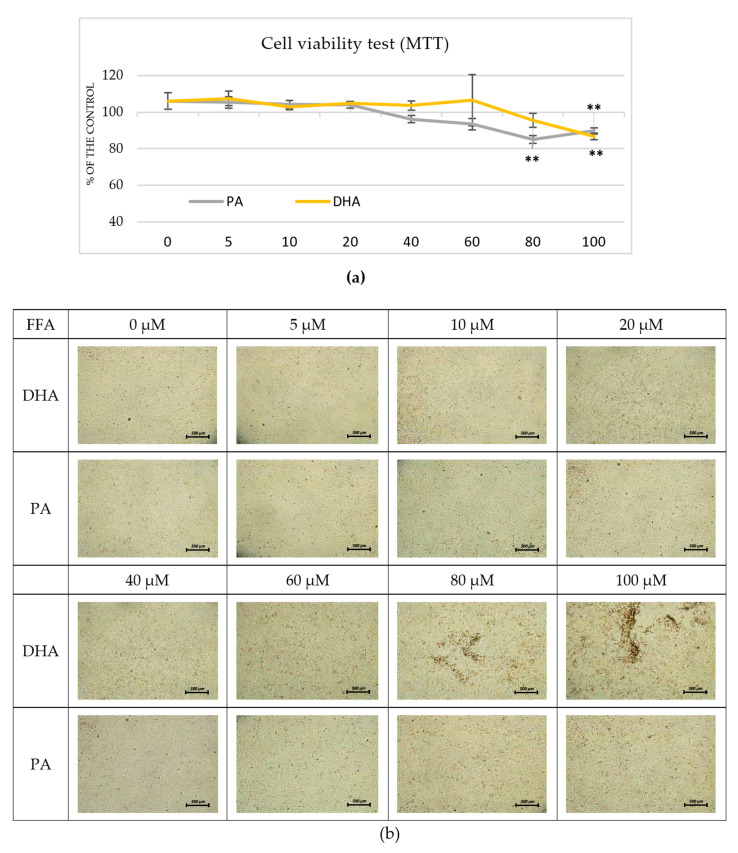
Effect of DHA and PA on cell viability in postconfluent 3T3-L1 cells (pre-experimental procedure). The postconfluent 3T3-L1 preadipocytes were treated with DHA or PA at various concentrations (0–100 μM) for 48 h. (**a**) An MTT assay was performed to evaluate cell viability. The data, expressed as a percentage of the control (non-treated preadipocytes), are presented as means ± SEM (*n* = 6). The statistical significance of differences was evaluated via a Mann–Whitney U test; ** indicates *p* < 0.01 compared to the control. (**b**) Native images of the 3T3-L1 cells were captured at 4× magnification (bars = 500 µm).

It is widely recognized that cells in the early stages of in vitro media-induced adipogenesis are highly susceptible to external factors. Applying free fatty acids (FFAs) to cell supernatants in inappropriate doses could easily reduce their viability during maturation. Thus, we first tested the effects of different concentrations of palmitic acid (PA) and DHA (5, 10, 20, 40, 60, 80, and 100 µM) on post-confluent 3T3-L1 preadipocytes and found that concentrations exceeding 60 µM reduced the cell viability (*p* < 0.01) in both treatments (Figure 1a). Upon exposure to 80 and 100 µM of DHA, the cells displayed significant morphological changes, including cell shrinkage and partial monolayer detachment (Figure 1b).

**Figure 2:**
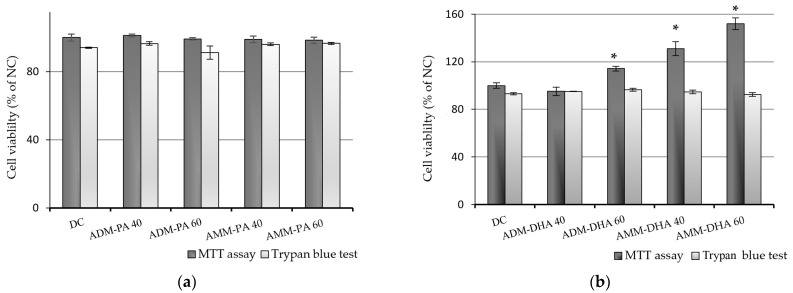
Cell viability of early-stage differentiating 3T3-L1 cells, exposed to either 40 or 60 μM of PA (**a**) or DHA (**b**) (pre-experimental procedure). Post-confluent 3T3-L1 cells underwent adipogenic induction over 4 days. Adipocyte differentiation medium (ADM) was administered during the first 2 days and then replaced with adipocyte maintenance medium (AMM) for the subsequent 2 days. PA or DHA was introduced to the culture media at 40 or 60 μM concentrations. Half of the cells were treated along with ADM (days 1 and 2), and the remaining half with AMM (days 3 and 4). Cell viability was assessed on day 4 of adipogenic differentiation, using the MTT assay and the Trypan blue test. The data, expressed as a percentage of the undifferentiated, non-treated group (NC), are presented as means ± SEM (*n* = 6). The statistical significance of the differences was evaluated via a Mann–Whitney U test; * *p* < 0.05—DC vs. each other group.

We further investigated if the maximum safe concentrations that have been established (40 and 60 µM PA/DHA) could induce a cytotoxic effect when applied with a specific culture media (adipocyte differentiation medium (ADM) or adipocyte maintenance medium (AMM)) on early-stage differentiating adipocytes. No effect on cell viability or cell number was observed when the differentiating growth-arrested 3T3-L1 preadipocytes were treated with 40 or 60 µM FFAs, regardless of the culture media used (Figure 2).

Based on the assays, we assumed that 60 µM DHA/PA was the maximum safe concentration in post-confluent and adipogenic differentiating 3T3-L1 cells.

### 2.2. The Effect of DHA on Adipogenesis

**Figure 3:**
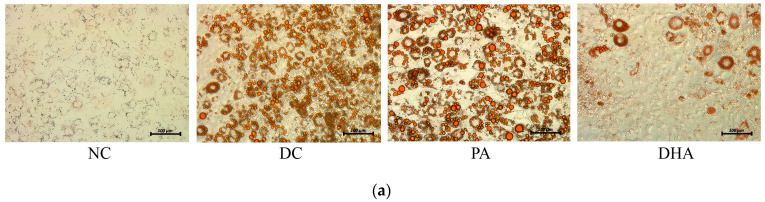

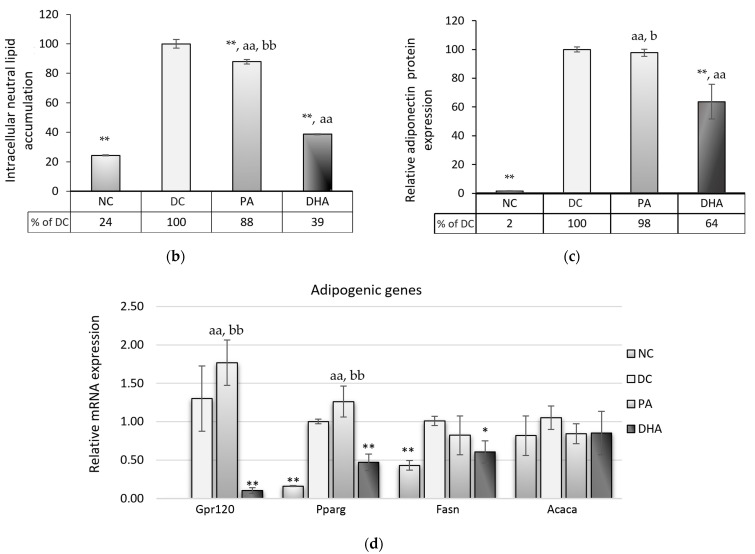
Inhibition of adipogenesis in preadipocytes by DHA. (**a**) Microscopic images from undifferentiated, non-treated (NC), differentiated, non-treated (DC), differentiated, and treated with PA (PA) or DHA (DHA) groups after Oil Red O staining were captured (20× magnification; bars = 100 µm). (**b**) Isopropanol extracts from the lipid fraction in each well were spectrophotometrically assessed at a wavelength of 490 nm. (**c**) Adiponectin protein expression was estimated in each supernatant using an ELISA. (**d**) The mRNA gene expression levels of adipogenic markers—including the G protein-coupled receptor 120 (Gpr120), peroxisome-proliferator-activated receptor gamma (Pparg), fatty acid synthase (Fasn), and acetyl-CoA carboxylase (Acaca)—were measured. The data, expressed as a percentage of DC, are presented as means ± SEM (*n* = 5). The statistical significance of differences was evaluated via a Mann–Whitney U test and is denoted as follows: *, b *p* < 0.05, **, aa, bb *p* < 0.01.

Nine days after the initiation of differentiation, we monitored the morphological changes in adipocytes stained with Oil Red O, measured the accumulation of neutral intracellular lipids, and analyzed the gene expression changes of Pparg, Gpr120, fatty acid synthase (Fasn), acetyl-CoA carboxylase (Acaca), and adiponectin protein concentration in the cell supernatants to estimate the effect of DHA supplementation on adipogenesis (Figure 3).

The images presented in Figure 3a demonstrate the advanced differentiation of adipocytes from DC and PA groups with well-defined intracellular lipid droplets. In contrast, primarily undifferentiated cells were observed in the DHA group, with only single cells being detected at the terminal differentiation phase. Most of the differentiated adipocytes in this group contained fine-grained lipid inclusions. Correspondingly, spectrophotometric measurements of the accumulated neutral lipids showed a significant increase of 76% in the DC group and 64% in the PA, as well as a slight increase of 15% in the DHA group compared to the NC (*p* < 0.01) (Figure 3b).

To elucidate the mechanisms underlying the observed lipid storage discrepancies among the groups, we assessed the gene expression of omega-3-sensitive adipogenic regulators: Pparg, a primary adipogenic initiator, and Gpr120, a specific modulator of the pathways that enhance fat storage and insulin sensitivity, and directly affect Pparg activity. The expression levels of both genes were elevated in well-differentiated groups (DC and PA) (*p* < 0.01) but were downregulated in the DHA compared to DC (*p* < 0.01), with no significant differences between the NC and DHA. Additionally, we evaluated the expression of late-stage adipogenic markers at the gene (Fasn) and protein (adiponectin) levels. As is consistent with previous findings, the increased expression of Fasn and adiponectin were established in the DC relative to the NC and DHA (*p* < 0.05, *p* < 0.01, respectively). Upon comparison between the NC and DHA groups, no significant difference was observed in Fasn gene expression levels. However, a statistically significant elevation in the release of adiponectin was noted in the DHA group (*p* < 0.01), corresponding to the extent of lipid accumulation.

Lastly, we explored the gene expression of Acaca, a rate-limiting enzyme in fatty acid synthesis that catalyzes the conversion of acetyl-CoA to malonyl-CoA. However, no significant differences among the groups were found.

Based on the aforementioned results, we can convincingly assert that the application of 60 µM DHA for 8 days at the onset of adipogenesis significantly suppresses intracellular lipid accumulation in 3T3-L1 adipocytes.

### 2.3. The Effect of DHA on Cell Number and Viability at the End of the Experiment

**Figure 4:**
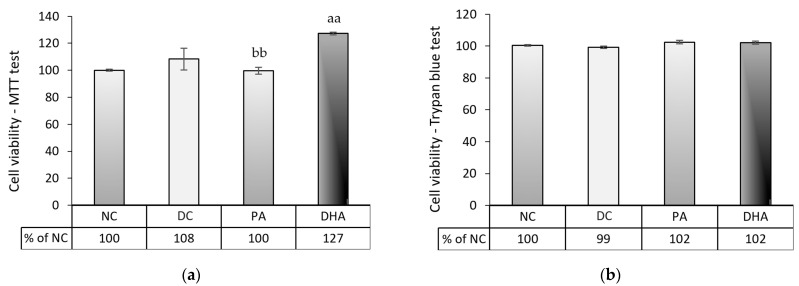
The cell viability of 3T3-L1 adipocytes exposed to DHA during the differentiation process was assessed via MTT assay (**a**) and by the Trypan blue test (**b**). Four groups were analyzed, including undifferentiated, non-treated (NC), differentiated, non-treated (DC), differentiated, and treated with PA (PA) or DHA (DHA). The data, expressed as a percentage of DC, are presented as means ± SEM (*n* = 5). The statistical significance of differences was evaluated via a Mann–Whitney U test and is denoted as follows: aa, bb *p* < 0.01.

To investigate whether the apparent inhibitory effect on adipogenesis observed in the DHA-treated group was caused by a decreased number or cell viability of adipocytes, we performed the MTT assay and Trypan blue exclusion test at the end of the experiment. The results of both tests, presented in Figure 4, demonstrated that after 8 days of 3T3-L1 adipocyte differentiation in a surplus of 60 µM DHA or PA, neither treatment resulted in an apoptotic effect. The DHA enhanced cell viability compared to the NC and PA groups (*p* < 0.01).

### 2.4. The Effect of DHA on Lipolysis

**Figure 5:**
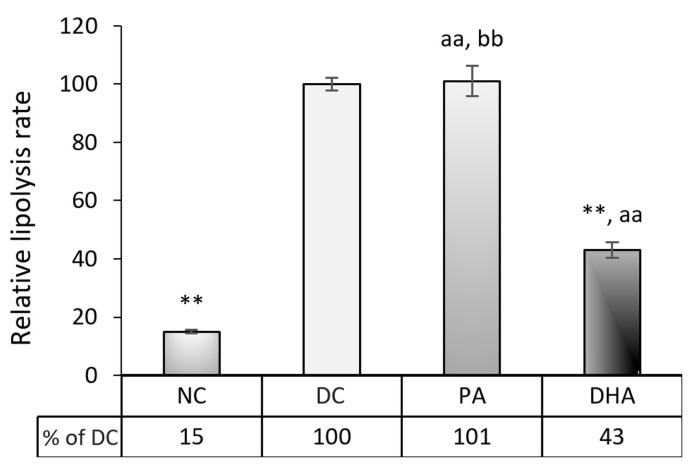
DHA suppresses the lipolysis rate in 3T3-L1 adipocytes. Four groups were analyzed, including undifferentiated, non-treated (NC), differentiated, non-treated (DC), differentiated, and treated with PA (PA) or DHA (DHA). The data, expressed as a percentage of DC, are presented as means ± SEM (*n* = 5). The statistical significance of differences was evaluated via a Mann–Whitney U test and is denoted as follows: **^,^ aa, bb *p* < 0.01.

In the next stage, we tested whether the suppression of adipogenesis in the DHA-treated group resulted from increased lipolysis. In the supernatants taken for testing at the end of the experimental period, we analyzed the level of lipolysis based on the glycerol released from the adipocytes. The lipolysis rate in the DHA group decreased by 57% compared to DC (*p* < 0.01) and slightly increased by 28% related to NC (*p* < 0.01) (Figure 5). Conversely, PA supplementation enhanced lipolysis by 85% compared to NC (*p* < 0.01) and maintained it at a level similar to DC.

These changes in the rate of lipolysis are in full agreement with the established levels of intracellular lipid accumulation.

### 2.5. Carnitine Palmitoyltransferase 1 (Cpt1) Gene Expression

**Figure 6:**
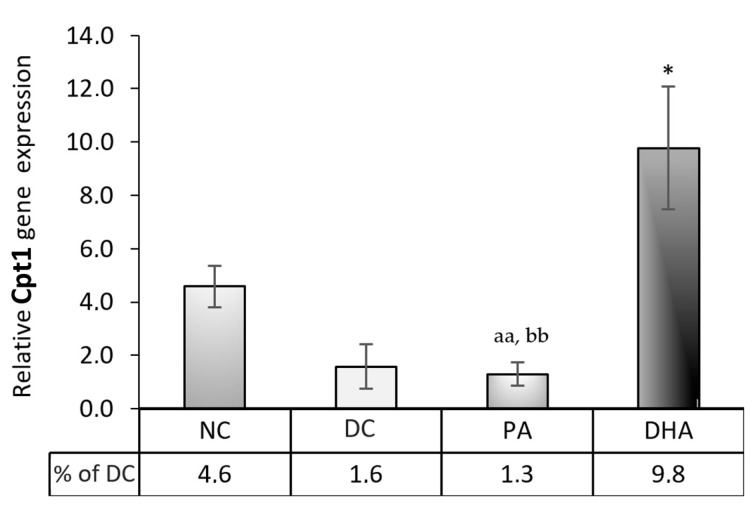
Relative mRNA gene expression of carnitine palmitoyltransferase 1 (Cpt1) in 3T3-L1 exposed to DHA. Four groups were analyzed, including undifferentiated, non-treated (NC), differentiated, non-treated (DC), differentiated, and treated with PA (PA) or DHA (DHA). The data, relatively expressed to DC, are presented as means ± SEM (*n* = 5). The statistical significance of differences was evaluated via a Mann–Whitney U test and is denoted as follows: * *p* < 0.05; and aa, bb *p* < 0.01.

To evaluate the influence of DHA supplementation on mitochondrial fatty acid β-oxidation, we analyzed the gene expression of a key rate-limiting enzyme in this process—Cpt1—and found a six-fold increase, compared to the DC (*p* < 0.05) and PA groups (*p* < 0.01) (Figure 6). It is likely that the increased glucose influx and intracellular lipid accumulation observed in the DHA compared to the NC group provide sufficient Cpt1 substrate. Therefore, the ultimate reduction seen in intracellular lipids may result from a complex interplay between the DHA-induced downregulation of adipogenic gene expression and a metabolic shift toward increased β-oxidation.

### 2.6. The Effect of DHA on Glucose Uptake

**Figure 7:**
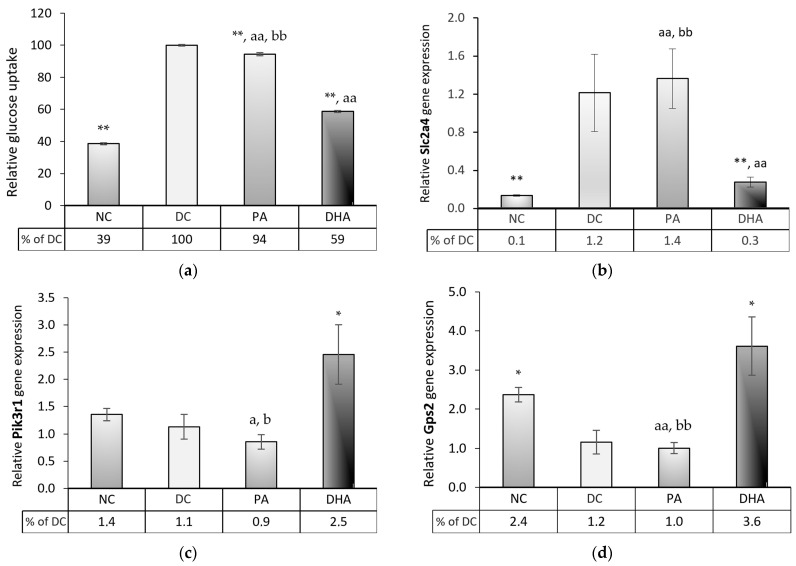
Glucose uptake and the mRNA gene expression level of critical insulin sensitivity modulators in 3T3-L1 adipocytes. Four groups were analyzed, including undifferentiated, non-treated (NC), differentiated, non-treated (DC), differentiated, and treated with PA (PA) or DHA (DHA). (**a**) Glucose uptake was measured in cell supernatants and the mRNA gene expression of (**b**) solute carrier family 2 member 4 (Slc2a4), (**c**) phosphoinositide-3-kinase regulatory subunit 1 (Pik3r1), and (**d**) G protein pathway suppressor 2 (Gps2) were assessed. The data, relatively expressed to DC, are presented as means ± SEM (*n* = 5). The statistical significance of differences was evaluated via a Mann–Whitney U test and is denoted as follows: *, a, b *p* < 0.05; and **, aa, bb *p* < 0.01.

To investigate DHA’s modulation of glucose uptake, we compared glucose levels in cell supernatants among the groups and examined the gene expression of solute carrier family 2 member 4 (Slc2a4), coding the glucose transporter 4 protein (GLUT4), which is responsible for rapid, insulin-dependent glucose influx in mature adipocytes. After 8 days of treatment during adipogenesis, the DHA group exhibited a 20% increase in glucose consumption compared to NC (*p* < 0.01) but a more than 35% decrease in uptake compared to the DC and PA groups (*p* < 0.01) (Figure 7a). These findings were consistent with the expression levels of Slc2a4 (Figure 7b), suggesting the inhibition of insulin-dependent glucose influx in adipocytes. We further studied phosphoinositide-3-kinase regulatory subunit 1 (Pik3r1) and Gps2, which are known for their antagonistic roles in activating the AKT signaling pathway, to acquire a more profound understanding of the molecular mechanism underlying their inhibitory effect on glucose uptake. Interestingly, our results indicated significant upregulation in both Pik3r1 (Figure 7c) and Gps2 (Figure 7d) gene expressions of the DHA-treated adipocytes compared to the DC and PA (*p* < 0.05). These results suggest that the inhibition of AKT membrane translocation may be a key mechanism underlying the reduced expression of Slc2a4 and the consequent inhibition of glucose uptake.

## 3. Discussion

The present research demonstrates that DHA application during 3T3-L1 preadipocyte differentiation suppresses adipogenesis and lipolysis, concomitantly decreasing glucose uptake. The obtained results closely resemble the findings from our previous investigation [34] and challenge the prevailing understanding that PUFAs act as agonists of Pparg in adipose tissue, thereby promoting adipogenesis through the enhancement of insulin-dependent glucose uptake and lipid deposition [15,35]. Indeed, several articles comparing the effects of various PUFAs demonstrate that DHA often outlines a specific mode of action and commonly alleviates intracellular lipid accumulation across diverse cell types [22,24,25,36,37,38]. However, whether this effect could be partially attributed to the induced apoptosis of adipocytes remains unclear. Kim et al. [23] have emphasized that the antiadipogenic effect of DHA in post-confluent 3T3-L1 preadipocytes is associated with direct cytotoxicity—the apoptotic effect after DHA administration was also established by other authors [24,25,39,40]. To rule out these possibilities, we assessed cell viability using the MTT and Trypan blue tests at the end of the treatment period. We demonstrated that none of the observed changes in the studied parameters were due to reduced cell numbers or decreased adipocyte viability.

Some authors have proposed that DHA supplementation exerts an anti-obesity effect by promoting lipolysis and upregulating essential lipolytic genes, such as adipose triglyceride lipase, hormone-sensitive lipase, and monoacylglycerol lipase [23,27,36]. Others claim that alleviated intracellular lipid accumulation upon DHA supplementation is mainly based on increased mitochondrial β-oxidation in adipose tissue [22,38]. Simultaneous studies, examining the effects of DHA in both preadipocytes and mature adipocytes, shed more light on these contrasting outcomes and underscore the importance of considering the stage of adipocyte maturation when developing anti-obesity strategies involving DHA modulation. The authors revealed that DHA enhanced lipolysis in mature adipocytes, while its impact on immature ones was either suppressive or insignificant [23,34]. We also found a notable reduction in lipolysis in response to DHA supplementation, accompanied by an upregulation of Cpt1 gene expression. These findings pinpointed DHA as a potent antiadipogenic agent that effectively redirects cellular metabolism in immature adipocytes toward mitochondrial oxidation and strongly mitigates a primary factor contributing to the onset of insulin resistance and lipotoxicity—the intensive release of free fatty acids. However, a significant concern arising from our results is that DHA-supplemented adipocytes exhibit a 40% reduction in glucose uptake, regardless of the high carbohydrate supply. This could be a decisive predisposing factor for a prediabetic condition in vivo.

To gain deeper insights into the intracellular mechanisms responsible for the observed metabolic alteration, we examined the expression of pivotal transcriptional-differentiation factors that are known for their pronounced sensitivity to PUFAs, namely, Pparg and Gpr120 [15,41,42,43]. Pparg is a crucial and irreplaceable regulator in adipogenic differentiation, with numerous Pparg targeting genes and epigenetic modulators being involved in this intricate process [35]. Recent studies have emphasized the membrane receptor Gpr120 as an essential contributor to adipogenesis, with its expression closely associated with that of Pparg [31,42]. Gpr120 directly affects Pparg activity by blocking its inhibitory phosphorylation, but any further expression upregulation of Gpr120 itself requires Pparg activation [31]. Therefore, Gpr120 is not detected in preadipocytes and is abundantly expressed in mature adipocytes [44,45]. Moreover, Gpr120 triggers distinct secondary messenger pathways, improving insulin sensitivity and facilitating healthy adipocyte expansion. As is consistent with these findings, we observed no expression of Gpr120 in the NC and its highest expression in the positive controls, DC and PA.

However, we determined a substantial downregulation of the Pparg and Gpr120 in the DHA-treated group compared to DC, accompanied by the suppression of late adipogenic markers, such as Fasn and adiponectin. Notably, matching the results between NC and DHA shows the lack of a significant difference in Pparg and Fasn gene expression and negligible upregulation in Gpr120. The observed alterations imply that prevalent cells exposed to DHA might not have initiated the adipogenic differentiation process, regardless of the potent inducers applied. This statement was further supported by the considerable proportion of undifferentiated adipocytes observed in the DHA group under microscopic examination.

Thus far, our data indicated that DHA supplementation mediated a specific intra-cellular mechanism involving suppressing Pparg transcription. Recent research has focused on the critical role of transcriptional co-regulators in determining adipocyte biology and metabolism. One such modulator seems to be the multifunctional subunit Gps2. It exhibits extensive genomic and non-genomic functions, simultaneously modulating the transcription of Pparg, mitochondrial function and biogenesis, glucose uptake, and insulin signaling, which emerged as a critical factor in facilitating physiological adipose tissue adaptation to high-energy intake [33,46,47]. Gps2 is a well-known NCoR/SMRT transcriptional co-repressor complex component in mammalian cells, and its activation suppresses the transcription of Pparg. The loss of Gps2 in preadipocytes enhances triglyceride accumulation and initiates adipose tissue differentiation by activating Pparg transcription [46,48,49]. However, its high expression in the early stages of adipogenesis would strongly inhibit adipogenic differentiation because, to date, no lipogenic inducer has been found to function independently of Pparg [35]. Accordingly, we established that Gps2 gene expression is inversely related to the adipocyte stage of maturity. It was downregulated in the DC and PA groups and remarkably upregulated in the DHA group, where the inhibition of adipocyte maturation was found.

We also observed an upregulation of Cpt1 gene expression in the DHA-treated group, suggesting enhanced mitochondrial fatty acid oxidation, which was further confirmed by the higher levels of formazan measured during the MTT viability test. Despite being relatively understudied in this direction, Gps2 is assumed to be also a potent modulator of mitochondrial function in adipocytes by adapting the expression of a critical signaling mediator of hypoxia response in growing adipose tissue—Hypoxia-inducible Factor 1α [32,47,48].

However, mature adipocytes that are deficient in Gps2 undergo maladaptive expansion, which is characterized by specific membrane remodeling, including sphingomyelin depletion, phosphatidylcholine, phosphatidylethanolamine enrichment, and collagen deposition [48]. This process also involves the reduction of phospholipids containing PUFAs and the accumulation of those that are rich in monounsaturated fatty acids, further contributing to the onset of insulin resistance, tissue inflammation, mitochondrial dysfunction, and enhanced lipolysis in fat cells [48,50]. Hence, it is unsurprising that we observed a significant inhibition of Pik3r1 gene expression in the DC and PA and its upregulation in DHA-treated adipocytes. This suggests an enhanced Pik3r1-AKT/Protein kinase B signaling pathway, ultimately leading to the activation and translocation of GLUT4 (Slc2a4) to the cell membrane. However, the established downregulation of Slc2a4 gene expression and the reduction in glucose consumption in this study contradicts the claim that DHA supplementation increases insulin sensitivity.

Cederquist et al. [50] have suggested that high levels of Gps2 in adipose tissue suppress insulin signaling by inhibiting ubiquitin-conjugating enzyme 13 (Ubc13) by Gps2 in adipocytes. This mechanism affects the Pik3r1-AKT signaling pathway by modulating the translocation of AKT/Protein kinase B to the plasma membrane. It is well known that the complete activation of AKT is promoted by dual phosphorylation through PDK1, a Pik3r1-dependent kinase, and the rictor-mTOR2 complex [51]. Notably, a rate-limiting event for activating this essential mediator in the insulin signaling pathway is not its phosphorylation per se but rather its translocation to the plasma membrane, which requires the ubiquitination of AKT, mediated by Ubc13 [50]. Chan et al. [52] have emphasized that the excessive stimulation of AKT can also lead to various negative consequences. AKT is not only involved in the insulin pathway but also serves as a crucial signaling molecule in the growth, survival, and metabolism of cells. Therefore, its overstimulation is directly linked to tumorigenesis in different cells [53,54]. Hence, maintaining physiological levels of Gps2 to regulate AKT activation within physiological norms is crucial for preserving the health status of adipose tissue that is exposed to high-energy intake.

The effects of Gps2 extend beyond adipocyte metabolism and have implications for insulin sensitivity at the organismal level. Other authors have reported improved β-cell function and muscle insulin sensitivity due to Gps2 upregulation [55]. In the context of enhanced systemic insulin signaling, the reduced glucose uptake resulting from suppressed adipocyte differentiation is not necessarily detrimental since glucose uptake can be preferentially redirected by the skeletal muscles, thus maintaining normoglycemia and preventing long-term complications.

The present study reveals that DHA has a clearly defined potential to prevent adipocyte differentiation and suppress lipolysis, regardless of the obesity-predisposing factors applied. However, whether the mechanisms underlying this effect could have a negative impact on overall health requires further investigation in vivo.

## 4. Materials and Methods

### 4.1. Cell Culture Handling

First, 3T3-L1 MBX clone mouse fibroblasts (3T3-L1) from the American Type Culture Collection (CRL-3242, ATCC, Washington, DC, USA) were propagated in T75. Then, they were seeded in 24- and 12-well plates (Corning, Costar) at a density of 10^4^/mL and grown in basal culture media (CM), composed of high-glucose Dulbecco’s Modified Eagle Medium (DMEM, 4.5 g/L glucose), 10% (*v*/*v*) fetal bovine serum (FBS) and 1% antibiotic solution (penicillin G, streptomycin and amphotericin B), all purchased from Sigma-Aldrich Chemie GmbH (Merck KGaA, Darmstadt, Germany).

The 3T3-L1 cell line was grown in a humidified atmosphere of 95% air with 5% CO_2_ and at 37 °C until reaching 100% confluency. Then, the cells underwent 24 h of growth arrest. Adipogenic differentiation was induced by applying adipogenic inductors for 48 h. The ADM consisted of CM supplemented with 10 µg/mL insulin (cell application, San Diego, CA, USA), 0.05 mM indomethacin (Sigma-Aldrich Chemie GmbH (Merck KGaA, Darmstadt, Germany)), 0.1 mM 3-isobutyl-1-methylxanthine (Cayman Chemical, Ann Arbor, ML, USA), and 1 µM dexamethasone (Sigma-Aldrich, St. Louis, MO, USA). Subsequently, the ADM was replaced by AMM, which contained CM and 10 µg/mL insulin alone, and the adipocytes were kept for an additional 7 days to reach full maturation.

### 4.2. Fatty Acid Dissolving Procedure

Docosahexaenoic acid (DHA, D2534, Sigma, Chemie, GmbH) and palmitic acid (PA, P5585, Sigma, Chemie, GmbH) were dissolved in 100% sterile, cell-culture-tested ethanol to create a stock solution, which was then stepwise diluted ex tempore in ADM or AMM to the desired final concentration of DHA/PA and 0.05% ethanol. Previously, we had ensured that ethanol concentration up to 0.1% (*v*/*v*) in the culture medium of 3T3-L1 cells did not influence their cell viability [56]. Therefore, we considered a final ethanol concentration of 0.05% safe for handling cells in the current investigation. The prepared stock solutions were kept at −20 °C until use.

### 4.3. Pre-Experimental Procedure: Cell Viability Assays

We seeded 3T3-L1 preadipocytes in 24-well plates at a density of 10^4^/mL and cultured them in CM until 100% confluence to determine the highest DHA and PA concentrations that were possible without cell viability issues. At this stage, the preadipocytes were exposed to 7 different DHA and PA concentrations for 48 h (5, 10, 20, 40, 60, 80, and 100 µM) with 6 wells per concentration. Pure 0.05% (*v*/*v*) ethanol in CM served as a control. Following a 48-h incubation period, morphological changes within each well were observed using a Leica inverted microscope outfitted with a 5-megapixel resolution DMi1 camera. Native images of each distinct treatment were captured at a four-fold magnification. The cellular activity from each concentration was assessed via the reduction of 3-(4,5-dimethyl-2-thiazolyl)-2,5-diphenyl-2H-tetrazolium bromide (MTT, Sigma-Aldrich, St. Louis, MO, USA) to formazan crystals, as described by Yang et al. [57] with some modifications. Briefly, 60 µL of MTT solution (5 mg/mL) was added to each well and incubated at 37 °C for 80 min. Then, the supernatants were replaced by the same volume of 0.04 N solution of HCl in isopropanol (Sigma-Aldrich Chemie GmbH (Merck KGaA, Darmstadt, Germany)) and shaken for 10 min to dissolve the formazan crystals. Finally, the optical density was assessed at a 570 nm wavelength (Synergy™ LX Multi-Mode Microplate Reader, BioTek Instruments, Inc., Santa Clara, CA, USA), and the percentage of cell viability was calculated using the following equation [58]:

Cell viability (%) = [OD570 (sample)/OD570 (control)] × 100(1)

It is assumed that during the early stages of in vitro adipogenesis, when the inducers are applied, the preadipocytes are at their most sensitive, and even small changes in the environment, such as negligible modifications in inducer concentration, could detach the cells from the flasks. Therefore, before starting the experiment, we tested the chosen concentration of the FFA during the first days of the adipogenic induction. We grew 3T3-L1 preadipocytes in 24-well plates until they reached 100% confluency. After 24 h of growth arrest, we began adipogenic differentiation for four days (2 days in ADM and 2 days in AMM), as half of the cells were treated along with ADM (days 1 and 2), and the rest were treated during days 3 and 4 (the FFAs were implemented with AMM).

On day 4, the cell viability of 6 wells from each treatment was estimated using the MTT assay, as described above, and the Trypan blue exclusion method. Determination of total and viable cell percentage was performed using Trypan blue staining of the cell population—a modified method described by Danesi et al. [59]. Briefly, cells from each well were washed twice with PBS (Merck KGaA, Darmstadt, Germany), incubated with trypsin for 5 min, then centrifuged for 5 min at 1000× *g* and resuspended in 1 mL of CM. Ten µL of the suspended cells were mixed with an equal volume of Trypan blue solution (0.4% final concentration) (Merck KGaA, Darmstadt, Germany). After 10 min of incubation at room temperature, the total number of cells, as well as the number of dead cells (stained blue), was determined with a hemocytometer and then calculated using the formula:

% Living cells = [1.00 − (number of cells stained blue/total number of cells)] × 100(2)

Finally, the cell viability was expressed as a percentage of the control (non-treated preadipocytes).

### 4.4. Experimental Design

As described above, the 3T3-L1 preadipocytes were seeded and differentiated in 12- and 24-well plates for 8 days (2 days in ADM and 6 days in AMM). At the onset of the adipogenic differentiation, the cells were divided into 4 groups: NC—undifferentiated, non-treated cells (cultured only in CM); DC—differentiated, non-treated adipocytes; DHA—differentiated and treated with DHA; PA—differentiated and treated with PA. The PA group served as an isoenergetic control, simulating in vitro chronic energy excess via co-delivery of high glucose and saturated fatty acid. PA was selected due to its identification by researchers as one of the most prevalent fatty acids in circulation in both animals and humans [60]. Simultaneously, it has been recognized as the most harmful saturated fatty acid concerning insulin sensitivity and glucose transport [61].

The culture media was changed every 2 days, and fatty acids were diluted ex tempore in ADM or AMM at a final concentration of 60 µM FFAs. The whole treatment lasted 8 days. On the ninth day since the beginning of the experiment, all samples intended for investigation were systematically collected.

At the end of the experiment, an MTT assay and a Trypan blue exclusion test were implemented to investigate whether 60 µM DHA or PA had any apoptotic effect on the 3T3-L1 adipocytes. The tests were conducted in 24-well plates with 6 biological replicates for each group, following the methods that have been described above.

### 4.5. Assessment of Neutral Lipid Accumulation

To investigate the phenotype of mature adipocytes from the various experimental groups, images were obtained after Oil Red O (Sigma-Aldrich, St. Louis, MO, USA) staining to visualize the intracellular lipid droplets. Subsequently, we extracted the dye with isopropanol and quantified the neutral lipid accumulation in adipocytes using the method described by Yang et al. [62]. The supernatants from the 12-well plates were removed, and the cells were washed 3 times with phosphate-buffered saline (PBS). Next, the adipocytes were fixed in 10% (*v*/*v*) neutral buffered formalin for 10 min, washed with isopropanol for 5 min, and stained for 30 min with a working solution of Oil Red O, prepared ex tempore. The stained wells were then kept in dd H_2_O and observed using a Leica Inverted Microscope with a 5-megapixel-resolution DMi1 camera. The accumulated Oil Red O dye in the adipocytes was extracted with 100% isopropanol for 10 min. The optical density of the extracts was assessed spectrophotometrically at a wavelength of 490 nm using a Synergy™ LX Multi-Mode Microplate Reader (BioTek Instruments, Inc., USA). Finally, the data were expressed relative to the DC as a percentage.

### 4.6. Lipolysis Rate Evaluation

The glycerol concentration was quantified on day 9 of the trial using an adipolysis assay kit (MAK313; Sigma-Aldrich, St. Louis, MO, USA) to compare the lipolysis rates among the studied groups. Each sample was measured twice, in an amount of 10 µL, according to the protocol provided by the manufacturer. Optical density (OD) at a 570 nm wavelength with a correction of 630 nm was measured (Synergy™ LX Multi-Mode Microplate Reader, BioTek Instruments, Inc., USA). Glycerol concentration was calculated in µg/mL according to the OD of the standards and the standard curve. Then, the percentage of lipolysis was assessed relative to DC.

### 4.7. Glucose Consumption Estimation

At the end of the experiment, we collected and analyzed the supernatants from the 12-well plates to determine the extracellular concentration of glucose (EG) using a glucose colorimetric assay kit (MAK013, Sigma-Aldrich, St. Louis, MO, USA). All samples and standards were duplicated and prepared for analyses according to the manufacturer’s instructions, and their final absorption was measured at 570 nm. Glucose concentration was calculated based on the OD of the standard sucrose curve and the background correction of the assay blank. Corresponding culture media from each group, without cells, were also maintained under the same conditions and analyzed in parallel with the experimental supernatants. The concentration of glucose in them was taken as the initial concentration (IG) and was then used to calculate glucose uptake, using the equation described by Diaz et al. [63]:

Glucose uptake (mmol/l) = IG − EG(3)

### 4.8. Gene Expression Analyses

The adipocytes from the 12-well plates were lysed, and the mRNA was isolated. The RNeasy Mini Lipid Tissue Kit (QIAGEN GmbH, Hilden, Germany) was used, following the methodology described by the manufacturer. The RNA quantity and quality were determined spectrophotometrically at 260 and 280 nm wavelengths using an Agilent Cary 60 UV/Vis device. After equilibrating the samples’ concentration, reverse transcription was conducted employing the RevertAid First Strand cDNA Synthesis Kit (Thermo Scientific, Waltham, MA, USA). The cDNAs were stored at −20 °C until use.

The primer pairs of the housekeeping (β-actin (Actb); hypoxanthine guanine phosphoribosyl transferase (Hprt), 18S ribosomal RNA (18S)) and target genes (Pparg, Gpr120, Fasn, Acaca, Cpt1, Slc2a4, Pik3r1, Gps2) were designed in Primer 3_4.1 with the NCBI software, according to the Luminaris HiGreen master mix manufacturer’s criteria for qPCR Master Mix (ThermoFisher). The specification of the primer pairs was further confirmed in Primer-BLAST (NCBI) https://www.ncbi.nlm.nih.gov/tools/primer-blast, Primer 3plus (version: 3.2.5) https://www.bioinformatics.nl/cgi-bin/primer3plus/primer3plus.cgi and SerialCloner (version 2.6.1), all accessed during the period from 22 April to 12 May 2022. The predicted products’ melt curves were analyzed using uMelt QuartzSM, accessed on 12 May 2023 (version: release: 3.6.2 “Quartz”/5 November 2020). The primer sequences are shown in Table 1. The 18S primer sequence was designed and cited by Arnhold et al. [64].

RT-qPCR analysis was performed, based on the SYBR RCR thermal cycler Gentier 96E (China), for the relative determination of the target genes’ expression with the already synthesized cDNA. The temperature program by the SYBR Green Real-Time PCR Master Mix’s manufacturer (Thermo Fisher Scientific, Waltham, USA), was followed.

The housekeeping genes’ stability was evaluated using the RefFinder software. All of the referent genes were analyzed separately and in different combinations. The results revealed that the average value of the 3 housekeeping genes (Actb, Hprt, and 18S) was the most stable in most of the implemented tools. Therefore, the effects concerning gene expressions were relatively estimated by applying the modified 2^−∆∆Ct^ approach with multiple reference genes [65,66].

### 4.9. ELISA Adiponectin Quantification

Adiponectin protein levels in the cell culture supernatants were assessed using the enzyme-linked immunosorbent assay (ELISA) kit, cat. no. RAB1115 (Sigma-Aldrich, Chemie GmbH (Merck KGaA, Darmstadt, Germany)). The samples were analyzed immediately after collection, following the sandwich assay procedure described by the manufacturer. The results were first estimated as adiponectin protein levels in pg/mL according to the OD of the standard curve. Then, the percentage of adiponectin protein expression was assessed, relative to DC.

### 4.10. Statistical Analysis

Statistical data analyses were conducted using the software system Statistica, version 10 (StatSoft Inc., 2011, Tulsa, OK, USA). Descriptive data analysis was applied to assess the mean and standard error of the mean. The nonparametric Mann–Whitney U test was employed to determine the significance of the differences between the control (NC/DC) and each of the other groups and to compare the effects of DHA with PA. As a minimal, statistically significant difference, *p* < 0.05 was accepted.

## 5. Conclusions

Our findings position DHA as a potent antiadipogenic agent in immature adipocytes that inhibits lipolysis, regardless of the strong differentiating inducers applied. This study also rules out cytotoxicity as a cause of these effects, even demonstrating increased cell viability. The gene expression analysis suggests that DHA hinders the onset of adipogenesis by suppressing Pparg, thereby downregulating critical pro-adipogenic genes. Additionally, DHA interactions are proposed to redirect cellular metabolism toward mitochondrial β-oxidation, further augmenting its antiadipogenic effect. Our study underscores the novel finding that Gps2 expression may play a pivotal role in mediating the DHA-induced intracellular signaling pathways’ modulation and emphasizes the importance of considering the adipocyte maturation stage in anti-obesity strategies. Notably, the observed reduction in glucose uptake in DHA-supplemented adipocytes raises concerns about potential adverse health effects. Therefore, further investigations in vivo are necessary to comprehensively elucidate the mechanisms by which DHA governs the outcomes revealed in this study and to gain a deeper understanding of its therapeutic potential for preventing obesity and obesity-related comorbidities.

## Figures and Tables

**Table 1:** Gene-specific primer sequences for real-time PCR analysis, with the corresponding PCR product sizes and gene accession numbers.

Abbreviation	Full Name	Forward Primer	Reverse Primer	Product Size (bp)
Pparg NM_001127330.2	Peroxisome proliferator-activated receptor gamma, transcript variant 2	AGGGCGATCTTGACAGGAAA	CGAAACTGGCACCCTTGAAA	164
Gpr120 NM_181748.2	G protein-coupled receptor 120	CCAACCGCATAGGAGAAATC	CAAGCTCAGCGTAAGCCTCT	140
Fasn NM_007988.3	Fatty acid synthase	CTGAAGCCGAACACCTCTGT	GGGAATGTTACACCTTGCTCCT	218
Acaca NM_133360.3	Acetyl-CoA carboxylase	TGCTCATGTTCCTTGCCCAA	TGCCACCACCATATTTGAGATT	247
Cpt1 NM_153679.2	Carnitine palmitoyltransferase 1	GTGTACTTCCAACTACGTCAGC	GCGATACAGGAGCAGGGTAT	169
Gps2 NM_001357906.2	G protein pathway suppressor 2	ACAAGTGCTTACGACCCGG	GGAAATGCTGATGGGGCTCT	183
Pik3r1 NM_001077495.2	Phosphoinositide-3-kinase regulatory subunit 1	ATACTTGATGTGGCTGACGC	GTCTCGCTTCCCTCGCAATA	193
Slc2a4 NM_009204.2	Solute carrier family 2 member 4	CGTTGGTCTCGGTGCTCTTA	AGCTCTGCCACAATGAACCA	220
Hprt NM_013556.2	Hypoxanthine guanine phosphoribosyl transferase	ACAGGCCAGACTTTGTTGGA	ACTTGCGCTCATCTTAGGCT	150
Actb NM_007393.5	β-actin	CCTCTATGCCAACACAGTGC	GTACTCCTGCTTGCTGATCC	211

## Data Availability

The datasets generated for this study are available from the corresponding author upon request.
